# A multiplex primer design algorithm for target amplification of continuous genomic regions

**DOI:** 10.1186/s12859-017-1716-7

**Published:** 2017-06-19

**Authors:** Ahmet Rasit Ozturk, Tolga Can

**Affiliations:** 1 Middle East Technical University, Informatics Institute, Ankara, Turkey; 2 Department of Computer Engineering, Middle East Technical University, Ankara, Turkey

**Keywords:** Next Generation sequencing, Target amplification, Multiplex PCR, Primer design

## Abstract

**Background:**

Targeted Next Generation Sequencing (NGS) assays are cost-efficient and reliable alternatives to Sanger sequencing. For sequencing of very large set of genes, the target enrichment approach is suitable. However, for smaller genomic regions, the target amplification method is more efficient than both the target enrichment method and Sanger sequencing. The major difficulty of the target amplification method is the preparation of amplicons, regarding required time, equipment, and labor. Multiplex PCR (MPCR) is a good solution for the mentioned problems.

**Results:**

We propose a novel method to design MPCR primers for a continuous genomic region, following the best practices of clinically reliable PCR design processes. On an experimental setup with 48 different combinations of factors, we have shown that multiple parameters might effect finding the first feasible solution. Increasing the length of the initial primer candidate selection sequence gives better results whereas waiting for a longer time to find the first feasible solution does not have a significant impact.

**Conclusions:**

We generated MPCR primer designs for the HBB whole gene, MEFV coding regions, and human exons between 2000 bp to 2100 bp-long. Our benchmarking experiments show that the proposed MPCR approach is able produce reliable NGS assay primers for a given sequence in a reasonable amount of time.

## Background

Advances in Next Generation Sequencing technologies decreased the cost-per-base below Sanger sequencing [1], leading to an increase for the demand of high-throughput and low cost NGS approaches [2]. Despite the overall high cost of Whole Genome Sequencing (WGS), targeted sequencing assays amplifying only selected regions of the genome are developed such as target amplification, target enrichment, and molecular inversion probes [3, 4]. Among the targeted sequencing approaches, targeted amplification method is more suitable for smaller genomic regions in order to get a uniform coverage and reliable read quality [3]. Median size of human exons is 120 bp and 70% of the human exons are shorter than 200 bp [5]. In this method, selected genomic regions are first amplified using PCR, then, PCR products are filtered and isolated, and sequenced with a NGS instrument [6]. A major drawback of the approach is the allele dropout, caused by a SNP in the 3′ end of a primer, resulting in low or no amount of expected PCR product. However, this problem can be overcome at the design level by including a primer-binding region in another PCR product [7]. In order to automate the process of amplification of a selected genomic region, special instruments, such as RainDance® are required [8]. A good alternative to achieve multiple amplification using conventional PCR is the Multiplex PCR (MPCR). For example, the consensus transcript of the MEFV gene (ENST00000219596) has 10 exons and 8 of them can be easily sequenced by popular desktop sequencers like Illumina MiSeq or Ion PGM instruments since the maximum length of the those exons is 357 bps. However the remaining 2nd and 10th exons are 633 and 554 bps, respectively. Since those lengths cannot be read in the desktop sequencers at once, they should be either amplified as shorter fragments or the whole exons should be fragmented using an experimental method, which results in additional experimental steps and more PCR experiments for those regions. However, a multiplex approach does not require additional experimental steps. In addition, costly PCR consumables like the polymerase enzyme are only used in a few tubes regardless of the number of fragments to be amplified. Therefore, sequencing cost of a small gene like the hemoglobin subunit beta

(HBB) and a larger one like the Mediterranean fever (MEFV) becomes almost the same.

The main limitation of the MPCR approach is the content of the gene itself. For a successful MPCR experiment, there should be as few secondary structures and dimers as possible whereas a feasible solution should be found among a very limited number of possible primer candidate sites. To our knowledge, a method for describing the design of MPCR primers for a continuous genomic region following best practices of reliable PCR design to be used in NGS does not exist. In this paper, a novel primer design method to amplify targeted genomic regions using a multiplex approach that is suitable to be used in NGS is proposed.

## Methods

### Problem definition

Theoretically, multiple targeted DNA regions can be amplified at the same time and this technique is called Multiplex PCR (MPCR) [9]. However, primer-primer interactions, primer-PCR product interactions, formation of inhibitory secondary structures, or thermodynamically favored side products prevent efficient amplification of multiple targeted DNA regions in the same tube. With careful consideration of possible interactions and their thermodynamic properties, it is possible to avoid these issues and conduct a successful MPCR experiment. At the center of solving the problems of MPCR is the design of primer oligonucleotide sequence regarding the concentrations of each molecule in the test tube.

### Constraints

- The length of a PCR product in current sequencing technologies acceptable for diagnostic use is usually limited to 500 bases. Also, for practical purposes, it should not be less than 300 bases.
- Primers should be long enough for a specific hybridization to the targeted genomic region, but it should not be very long in order to reduce the cost of production and secondary structure formation tendency. The interval of primer length should be limited to 23 to 30 bps for optimum length.
- To avoid non-specific PCR products, designed primers should only bind to the target region and nowhere else. Thus, each designed primer should be checked for alternative binding regions through a BLAST search against the targeted genome.
- Variations in the DNA sequence of individuals are heterogeneous in terms of type and genomic location. An unexpected variation in the last 3 bases of a primer results in a weakened binding of the primer to its target region in the DNA template, resulting in the formation of low PCR product concentration. Therefore, there should not be a known variation in the last three bases of a designed primer.
- Total number of G and C bases divided by the total length of an oligonucleotide gives the GC rate of given sequence. Optimum GC rate of a primer is 50%, and it should not be more than 70% or less than 30%.
- Secondary structure formation inhibits PCR and decreases the yield of PCR products. Thus, it should be avoided when possible. Interactions between primers (either homo or heterodimers) and hairpins (self-hybridization of an oligonucleotide forming a loop structure) should not be thermodynamically favored, and their ΔG value should be less than −3 Kcal.
- Melting temperature (Tm) is defined as the ideal temperature for the formation of a stable primer-DNA template complex. Tm of each designed primer should be very close to each other, within a difference of 0.5 °C, and each primer Tm should be within 0.5 °C of the specified optimum Tm.
- There should not be 4 bp-long or longer homopolymers in the primer.
- Due to a phenomenon called allele-dropout resulted by variations in the 3′ end of a primer, each primer region should be included in another PCR product except the first and last primers for the targeted whole DNA fragment. Therefore, MPCR primer pairs should be split into at least two test tubes so that there should be no overlapping and undesirable primer products in the same test tube.

### Formulation of the MPCR design problem as a graph problem

The MPCR primer design problem can be formulated as a graph problem, with primer pairs meeting the primer design criteria as nodes in the graph and with edges between two primer pairs if they meet the interaction constraints. Among a set of feasible candidate primer pairs, a subset meeting the requirements of a complete graph can be placed in the same test tube. For a successful design, there should be at least two or more complete graphs where their PCR products meet the constraints and cover the targeted DNA region.

This problem corresponds to finding a clique in the graph with a varying size and is an NP-Complete problem described by Downey (1995). The solution time to find the best primer pair design is exponential with respect to the target region length, and there are no known efficient solutions for this problem. Therefore, a depth-first heuristic approach is implemented to find the first solution that meets the given constraints since all optimum solutions meeting the criteria are experimentally acceptable.

### The proposed method

Regarding the problem definition and constraints, finding suitable primer pairs is a tree search problem in the space of feasible primer pairs. Due to the exponential complexity of the problem, a depth-first approach is favored to find an acceptable solution within reasonable amount of time. The rules for designing primer pairs are given as follows:

- Leftmost forward primer should be in the first *n* bases of the given sequence
- Position of the rightmost reverse primer should be in the last *n* bases of the given sequence.
- Next PCR product should be in a different test tube
- Pos(Forward_tube n mod m, k_) < Pos(Reverse_tube n-1 mod m, k_)
- Pos(Forward_tube n mod m, k_) > Pos(Reverse_tube n-2 mod m, k_)
- Pos(Reverse_tube n mod m, k_) > Pos(Reverse_tube n-1 mod m, k_)
- Pos(Forward_tube n mod m, k_) > Pos(Reverse_tube n mod m, k-1_)

where

- Pos(Forward _tube n mod m, k_) denotes the position of the first base of the *k*-th forward primer in the test tube *n* regarding a total of *m* test tubes and
- Pos(Reverse _tube n mod m, k_) denotes the position of the last base of the *k*-th reverse primer in the test tube *n* regarding a total of *m* test tubes

### Sample data and implementation of the method

Human exon sequences with a length of 2000 to 2100 bases are selected using the Ensembl BioMart MartView interface including upstream and downstream flanking sequences, 240 bases for each. The proposed method is implemented with a heuristic approach: since BLAST queries takes a significant time, candidate primer sequences from the selected exons are queried through BLAST before the test case and results are loaded into a local database. If there are more than one BLAST result having an E-value less than 0.01, those candidate primers are discarded.

In the test, the duration of the first feasible solution is recorded. Three factors are evaluated: 1) the order of candidate primers in terms of base position for a given sequence interval, 2) the effect of initial primer candidate sequence length, since it changes the number of starting forward primer candidates, either 120 or 240 bases, and 3) the time limit required to find a feasible solution, either for 240 or 480 seconds.

The test is conducted on a Mid 2010 iMac Computer with 2.93 GHz Inter Core i7 CPU and 16 GB 1333 MHz DDR3 RAM.

## Results

The effectiveness of a multiplex target amplification experiment depends on the following factors: 1) avoiding undesired secondary structure formation, 2) uniformity of melting temperature (Tm) of primers, 3) GC content of primers, 4) avoiding single nucleotide polymorphisms (SNPs) in the 3′ end of primers, and 5) uniqueness of genomic regions which would reduce non-specific binding of the primers to other regions other than the target site. The proposed method takes these factors into account and designs robust primers for given target sites. Although all of the factors can be calculated using specific algorithms, finding an acceptable solution depends mostly on the primers in initial primer candidate set, which are derived from the flanking region just before the targeted exon. Another factor that might effect the performance is the selection order of candidate primers for a given sequence interval. For example, using a forward primer very close to the targeted exon might result in lower number of tubes and less primer pairs whereas selecting the forward primer at the beginning of a flanking region might increase the number of pairs, which will increase in the complexity of finding compatible primer pairs.

In order analyze the factors that effect the performance of the method, 48 different test cases are evaluated for 3 different initial sequence and duration constraints, 4 different primer candidate order selection constraints, and 4 different numbers of multiplex tubes from 2 to 5. The experimental design is depicted in Table 1. Fisher’s exact test is utilized to assess the significance of differences between groups.

**Table 1:** Test groups for the evaluation of the proposed methods

	Short240: 240 s time limit with short initial candidate sequence (120 bases)	Short480: 480 s time limit with short initial candidate sequence (120 bases)	Long240: 240 s time limit with long initial candidate sequence (240 bases)
bothNormal: P_forward_ and P_reverse_ in normal order by base location	1. test group for 2 to 5 tubes	2. test group for 2 to 5 tubes	3. test group for 2 to 5 tubes
fwdReverse: P_forward_ in reverse order and P_reverse_ in normal order	4. test group for 2 to 5 tubes	5. test group for 2 to 5 tubes	6. test group for 2 to 5 tubes
revReverse: P_forward_ in normal order and P_reverse_ in reverse order	7. test group for 2 to 5 tubes	8. test group for 2 to 5 tubes	9. test group for 2 to 5 tubes
bothReverse: P_forward_ and P_reverse_ in reverse order	10. test group for 2 to 5 tubes	11. test group for 2 to 5 tubes	12. test group for 2 to 5 tubes

**Fig. 1:**
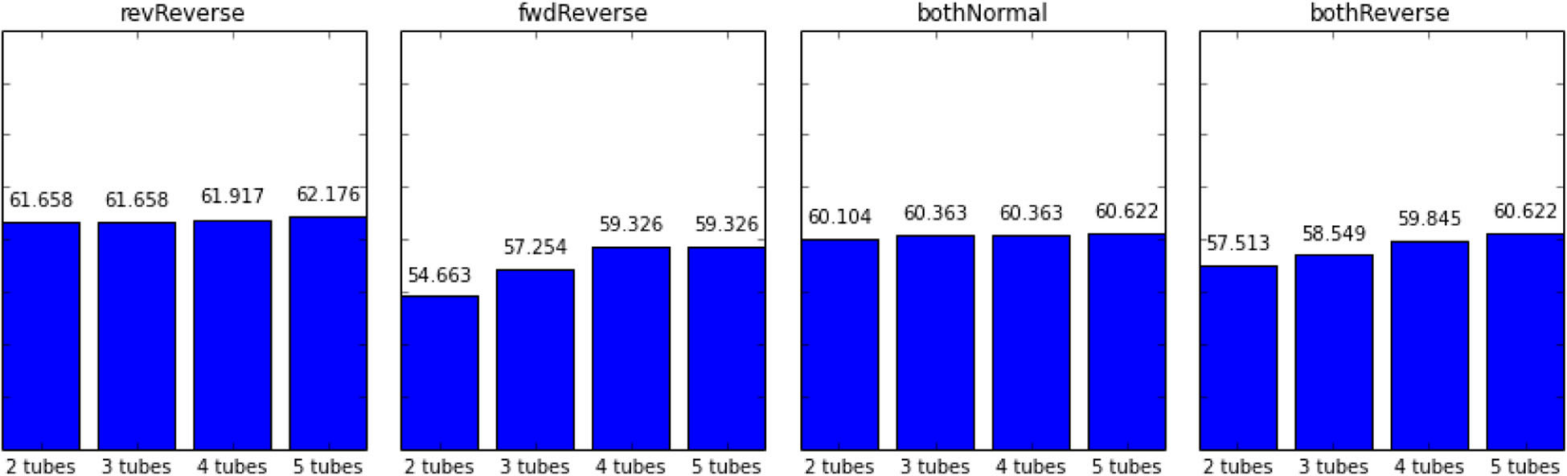
Upstream 120 bp of targeted regions are utilized as the first forward primer design space whereas downstream 120 bp are selected as the last reverse primer design region. Percentages of successfully designing MPCR primers for selected regions in 240 s with different candidate selection order approaches are shown

**Fig. 2:**
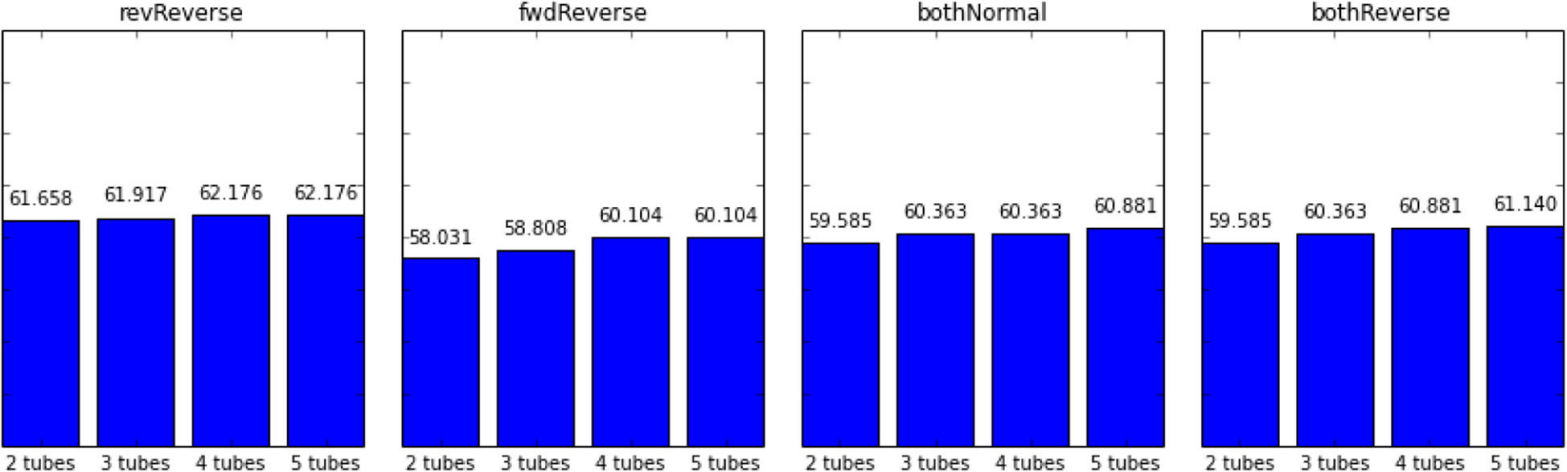
Upstream 120 bp of targeted regions are utilized as the first forward primer design space whereas downstream 120 bp are selected as the last reverse primer design region. Percentages of successfully designing MPCR primers for selected regions in 480 s with different candidate selection order approaches are shown

**Fig. 3:**
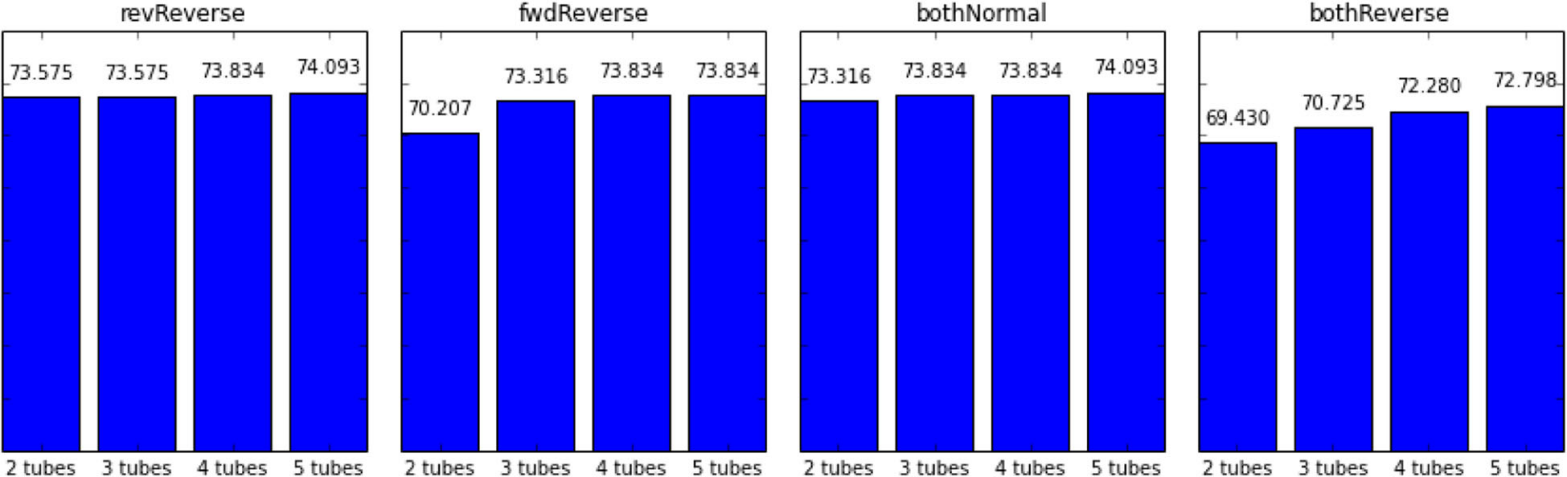
Upstream 240 bp of targeted regions are utilized as the first forward primer design space whereas downstream 240 bp are selected as the last reverse primer design region. Percentages of successfully designing MPCR primers for selected regions in 240 s with different candidate selection order approaches are shown

During the test, not all of the given situations resulted in a feasible solution within the limited time. However, success rates show differences in each case. Success rates for Long240, Short240 and Short480 test batches are shown in Figs. 1, 2 and 3, respectively.

Figures 1 and 2 show that increasing the time limit does not increase the success rate significantly (*p*-value = 1). However, Fig. 3 clearly shows that increasing the initial primer candidate sequence length have a dramatic effect on success rates (*p*-value = 0.033) since the initial primer candidate space harshly restricts the space of overall feasible solutions.

**Fig. 4:**
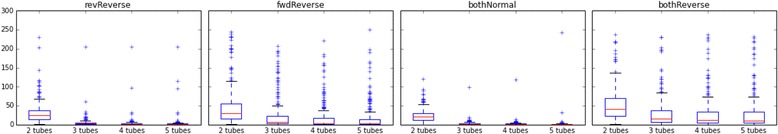
Upstream 120 bp of targeted regions are utilized as the first forward primer design space whereas downstream 120 bp are selected as the last reverse primer design region. Durations of successfully designing MPCR primers for selected regions in 240 s with different candidate selection order approaches are shown in seconds

**Fig. 5:**
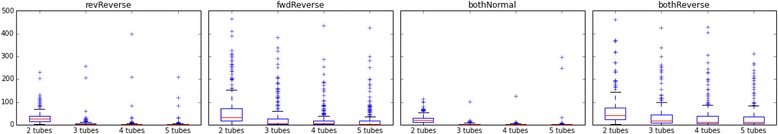
Upstream 120 bp of targeted regions are utilized as the first forward primer design space whereas downstream 120 bp are selected as the last reverse primer design region. Durations of successfully designing MPCR primers for selected regions in 480 s with different candidate selection order approaches are shown in seconds

**Fig. 6:**
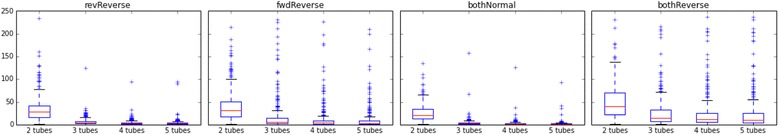
Upstream 240 bp of targeted regions are utilized as the first forward primer design space whereas downstream 240 bp are selected as the last reverse primer design region. Durations of successfully designing MPCR primers for selected regions in 240 s with different candidate selection order approaches are shown in seconds

The number of multiplex tubes used is another restriction on getting more successful solutions in limited time. In all test case groups, 2-tubes per amplification has the worst success rates (Figs. 1, 2 and 3). However, increasing the number of tubes from 3 to 5 does not have a significant time gain to get the first feasible solution for revReverse and bothNormal test cases (Figs. 4, 5 and 6) (*p*-value = 0.299 and *p*-value = 0.545, respectively).

**Fig. 7:**
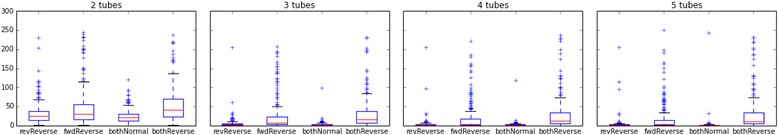
Upstream 120 bp of targeted regions are utilized as the first forward primer design space whereas downstream 120 bp are selected as the last reverse primer design region. Durations of successfully designing MPCR primers for selected regions in 240 s with different number of tubes are shown in seconds

**Fig. 8:**
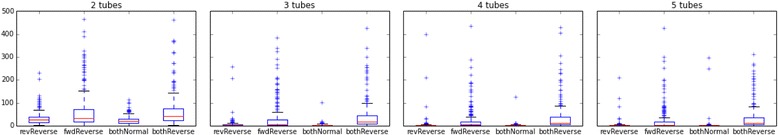
Upstream 120 bp of targeted regions are utilized as the first forward primer design space whereas downstream 120 bp are selected as the last reverse primer design region. Durations of successfully designing MPCR primers for selected regions in 480 s with different number of tubes are shown in seconds

**Fig. 9:**
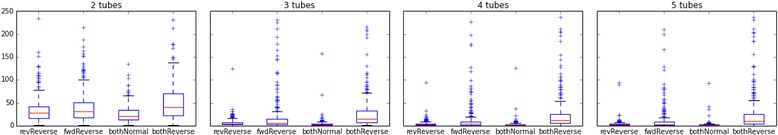
Upstream 240 bp of targeted regions are utilized as the first forward primer design space whereas downstream 240 bp are selected as the last reverse primer design region. Durations of successfully designing MPCR primers for selected regions in 240 s with different number of tubes are shown in seconds

Regarding the order of primer candidate selection each time for the same candidate sequence area, there are different factors that effect the performance of the method. revReverse and bothNormal test cases provide favorable results compared to fwdReverse and bothReverse test cases in all tests (Figs. 7, 8 and 9).

**Fig. 10:**
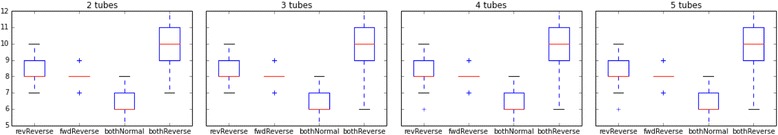
Upstream 120 bp of targeted regions are utilized as the first forward primer design space whereas downstream 120 bp are selected as the last reverse primer design region. Numbers of multiplex primer pairs for the first feasible solution in 240 s are shown as grouped by tube number

**Fig. 11:**
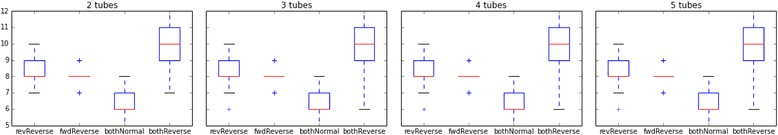
Upstream 120 bp of targeted regions are utilized as the first forward primer design space whereas downstream 120 bp are selected as the last reverse primer design region. Numbers of multiplex primer pairs for the first feasible solution in 480 s are shown as grouped by tube number

**Fig. 12:**
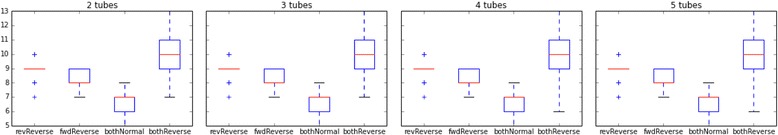
Upstream 240 bp of targeted regions are utilized as the first forward primer design space whereas downstream 240 bp are selected as the last reverse primer design region. Numbers of multiplex primer pairs for the first feasible solution in 240 s are shown as grouped by tube number

Lastly, it is observed that the number of primer pairs found for each multiplex primer solution is also affected by the order of candidate primer selection. bothNormal primer candidate selection order provides the lowest number of primer pairs for each solution, regardless of the number of tubes, time limit, or initial sequence length (Figs. 10, 11 and 12).

**Fig. 13:**
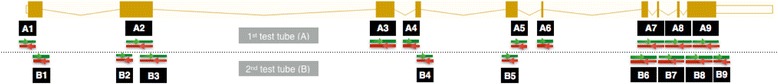
Distribution of MPCR primer pairs designed for MEFV transcript (ENST00000219596.5)

In addition, MPCR primers for coding regions of MEFV gene are designed using the proposed approach. Due to the short lengths of introns between the last four exons of MEFV transcript (ENST00000219596.5), that region should be considered as a single continuous DNA fragment for a feasible MPCR primer design which makes that genomic region an excellent use case of the developed algorithm. 18 primer pairs are designed as a result and seven of them cover the last four exons of the transcript (Fig. 13).

## Discussion

Due to practical reasons, benchmarking is limited with sequences between 2000 to 2100 bps long and with two different flanking sequence alternatives of either 120 or 240 bps. In addition, time to wait for the first feasible solution is limited to either 240 or 480 s. Although these settings clearly show the effect of changing the flanking sequence length and waiting time, a different setting with longer flanking sequence alternatives would increase the first set of primer candidates which in fact is the major factor of filtering out further primer candidates that are not thermodynamically compatible with the previous ones. Although selected sequences are human exons, the method can be applied to other organism to show the potential of the approach to be used for comparative genome studies. Lastly, the utility of the algorithm is shown on a real world case of MEFV transcript.

## Conclusions

Multiplex PCR is a convenient method for targeted NGS studies in terms of consumable cost, labor cost, and labor time compared to conventional PCR when amplifying multiple DNA fragments at the same time. However, due to the restrictions of primer design and complex primer-primer interactions, the problem reduces to an optimum subset clique finding problem in the network of all possible forward and reverse primer candidate sequences, which is an NP-complete problem [10]. Thus, finding the first feasible solution is an acceptable heuristic in regards to the nature of the problem.

On an experimental setup with 48 different combinations of factors, we have shown that multiple parameters might effect finding the first feasible solution. Increasing the length of the initial primer candidate selection sequence gives better results whereas waiting for a longer time to find the first feasible solution does not have a significant impact. Designing multiplex primers for 2 tubes is a more time-consuming problem than 3 tubes, but it does not increase dramatically when the number of tubes is increased from 3 to 5. Lastly, the selection order of candidate primers for a given sequence interval effects the duration of finding the first feasible solution as well as the number of primer pairs in a multiplex design solution. Selecting the candidate primers in normal order with regards to the increasing base location gives the best results in terms of both getting the lowest number of primer pairs and shortest duration for the first feasible solution. Multiplex primers for the HBB whole gene is designed using the proposed algorithm for 2 tubes. The algorithm is also applied for MEFV transcript and MPCR primers are successfully designed.

## Abbreviations

MPCR: Multiplex Primer Chain Reaction
NGS: Next Generation Sequencing
NP: Non-polynomial.
